# COVID-19 outbreaks in acute and long-term care: Conceptualizing patterns of vulnerability and benefits of interventions

**DOI:** 10.1016/j.lanepe.2022.100312

**Published:** 2022-02-01

**Authors:** Melissa K. Andrew, Shelly A. McNeil

**Affiliations:** a Department of Medicine (Geriatrics), Dalhousie University, Halifax, Canada; b Canadian Center for Vaccinology, Dalhousie University, Halifax, Canada; c Department of Medicine (Infectious Diseases), Dalhousie University, Halifax, Canada

SARS-CoV-2 outbreaks in acute and Long-Term Care (LTC) settings have taken a great toll, both in terms of morbidity and mortality from associated COVID-19 cases, and due to the dramatic impact on provision of other health and social services.^1^^,^^2^ Institutional outbreaks, and efforts to control and prevent them, have thus been important drivers of policy responses. As policies are implemented and evaluated, it is clear that we must aim to understand their incremental benefits and impacts to inform planning for ongoing pandemic response efforts.

In *The Lancet Regional Health Europe*, Suwono and colleagues examine SARS-CoV-2 outbreaks in hospital and long-term care facility settings in Germany, across four pandemic waves.^3^ The authors used mandatory notification data on SARS-CoV-2 cases in Germany and identified hospital and LTC outbreak cases within these. They calculated the correlation of outbreaks and outbreak cases with all cases in Germany in each pandemic wave. Then, in order to gain an understanding of the impact of non-pharmacological control measures (initiated in wave one [W1], and impacting all subsequent waves) and then vaccination (implemented at the end of W2, and chiefly impacting waves 3 and 4), they modeled counterfactual scenarios to calculate the number of outbreaks and cases averted in subsequent waves, with the aim of attributing the benefits back to this step-wise introduction of control measures.

They found that of the over four million COVID-19 cases to date in Germany, acute and LTC outbreaks accounted for approximately 1% and 4% of cases respectively. Overall, in pandemic W1 and W2 they observed a strong association in both hospital and LTC settings between outbreak cases and the total number of cases (all R>=0.93, p<0.001). However in W3 and W4, these correlations were weaker and the number of outbreaks and cases were much lower even in the face of substantial waves of community SARS-CoV-2 circulation (e.g. W3 R=0.38 acute and 0.66 LTC p<0.01; the slopes also flattened in W3&4). In their thoughtful discussion, the authors attribute the reduction from W1 to W2 to nonpharmacological control measures and the much more dramatic reductions seen in W3 and W4 to the addition of vaccination programs with high uptake in populations at risk. Using counterfactual models based on W1 conditions, they estimate that 53% of predicted outbreaks and 38% of cases in acute care were averted in W2 (51%/20% for LTC). In W3, 73% of outbreaks and 84% of cases were prevented in acute care, compared with 86% and 92% in LTC. A similar to even higher % outbreak and case avoidance was estimated for W4. Outbreak size was smaller in hospitals (overall median <=5 *vs*. >10 in LTC, though outbreak size was stable in acute care but showed substantial decreases in LTC outbreak size between successive waves), and they discuss how this could reflect differences in resources and staff training between settings. The weekly #LTC outbreaks was highly associated with cumulative uptake in vaccines among LTCF residents aged 65+ (R=-0.93, p<0.001).

Suwono *et al*’s paper is of particular interest because of the authors’ aim to tease out the impacts of both non-pharmacological measures and vaccines, benefitting from the sequential timelines in which they were introduced. The use of counterfactuals to estimate the number of outbreaks and cases prevented provides an accessible means of communicating the benefits of these important control measures which have sometimes proven difficult and burdensome (eg isolation resulting from visiting restrictions, vaccine hesitancy in the face of workplace mandates).^4^, ^5^, ^6^, ^7^, ^8^ Conducting this research in a real-world setting brings benefits of relevance and embracing complexity along with limitations stemming from the fact that it is hard to fully account for this complexity. To address these challenges, Suwono *et al.* employ creative and rigorous modeling approaches, including counterfactuals (to demonstrate benefits in real terms of outbreaks and cases prevented) and sensitivity analyses to test their assumptions. These types of analyses are an important tool in our pandemic toolbox to understand prevention while shaping messaging and actions going forward.

The Suwono *et al.* study also gives us the opportunity to consider the burden of and importance of preventing facility-level SARS-CoV-2 outbreaks, while bringing to light differing susceptibilities in acute *vs*. LTC settings. In both settings, an ecological approach can help conceptualize the many levels of vulnerabilities, from individual-level frailty and comorbidity, to shared living and care environments, resourcing and staffing constraints, and even the value placed by society on the people and settings themselves.^9^ The findings show that although outbreaks occur in a context, there are ways we can prevent them having the same spikes as in the circulating community. The institutional setting presents characteristics that can facilitate spread in outbreaks, but also offers the opportunity to protect those who are vulnerable through good policy and actions, both proactive and reactive. Interestingly, although the absolute performance appears better in hospitals, with smaller outbreak sizes, given the frailty of residents and specific susceptibilities in the LTC setting, it is notable that the LTC setting had the most consistent improvements over time as control measures were implemented and refined.

All in all, the Suwono *et al.* paper demonstrates the benefits of control measures in the current pandemic, while considering lessons for other future institutional outbreaks. One important message is that high vaccination uptake has added a key protective shield, which will hopefully allow adaptation of other control measures to better balance quality of life and prevent social isolation in these settings. We must also remember the ongoing benefits of strong, well-resourced, and responsive Infection Prevention and Control efforts. Hopefully this message serves to bolster immunization coverage efforts and inspire continued development of vaccines against this and other pathogens of outbreak potential, with a particular view to tailoring their design to optimize immune responses in older and high-risk individuals.^10^

## Declaration of interests

MKA reports grants from the Canadian Frailty Network, CIHR, Public Health Agency of Canada, Sanofi, Pfizer, Merck and GSK, and payments from Pfizer, Sanofi and Seqirus outside the submitted work. SAM reports grants and payments from Pfizer, GSK, Merck, Novartis and Sanofi, outside the submitted work.
